# Aerosols, airflow, and airspace contamination during laparoscopy

**DOI:** 10.1093/bjs/znab114

**Published:** 2021-04-08

**Authors:** N Hardy, J Dalli, M F Khan, K Nolan, R A Cahill

**Affiliations:** 1 UCD Centre of Precision Surgery, School of Medicine, University College Dublin, Dublin, Ireland; 2 School of Mechanical and Material Engineering, University College Dublin, Dublin, Ireland; 3 Department of Surgery, Mater Misericordiae University Hospital, Dublin, Ireland

## Abstract

Laparoscopic surgery has been undermined throughout the COVID-19 pandemic by concerns that it may generate an infectious risk to the operating team through aerosolization of peritoneal particles. There is anyway a need for increased awareness and understanding of the occupational hazard for surgical teams regarding unfiltered escape of pollutants generated by surgical smoke and other microbials. Here, the aerosol-generating nature of this access modality was confirmed through repeatable real-time methodology both qualitatively and quantitively to inform best practice and additional engineering solutions to optimize the operating room environment.

## Introduction

The COVID-19 pandemic has focused attention on the infectious transmission risk of laparoscopy for operating room (OR) staff^1^^,^^2^. In addition, there needs to be increased understanding of the potential dangers from surgical smoke pollution in surgical theatres despite positive-pressure room ventilation; this issue is now being prioritized by the Joint Commission^3^ among other groups^4^^,^^5^. Better empirical understanding of aerosols, airflow impact, and airspace contamination of laparoscopy would inform best practice as well as its appropriateness for classification among aerosol-generating procedures, while promoting and inspiring methods for hazard mitigation. Here, the methodology for such advancement is described, along with early findings from such evaluations including during surgery^6^^,^^7^.

## Methods

With institutional ethics approval (AEROSOLVE study, institutional review board reference 1/378/2172) and individual participant consent (from both OR team members and, when involved, patients), flow visualization studies were performed in the OR before and during elective laparoscopic operations. First, formal smoke studies were used to detail room ventilation dynamics around the operating table during surgical simulation scenarios with and without positive-pressure room ventilation (25 room air exchanges per h). For this, an Air-Trace smoke generator (Concept Engineering, Maidenhead, UK) created low levels of isokinetic, isothermal smoke via a 25-mm duct. The scenarios replicated personnel and equipment conditions for surgical procedures with varying complexity of set-up (open inguinal hernia repair, laparotomy, laparoscopic appendicectomy, laparoscopic cholecystectomy, and laparoscopic anterior resection) with the smoke generator hose positioned at the simulated operative site.

Thereafter, flow studies were performed during actual elective operations with varying degrees of intraoperative electrocautery (6 procedures; 3 laparoscopic cholecystectomies, 2 laparoscopic appendicectomies, 1 laparoscopic parastomal hernia repair; standard pneumoperitoneal pressure setting 12 mmHg). For this, a light sheet generated by a galvanometer optical laser scanner was used to illuminate a two-dimensional (2D) slice of the surgical airspace during surgery and imaged with an 8 K Ultra-High Definition camera (Canon EOS R5 with RF 35 mm f/1.8 lens; Canon, Ohta-ku, Tokyo, Japan) whose absence of diffraction limitation enabled resolution of droplets larger than 2 µm (visible as scintillations within the laser sheet). Simultaneous extracorporeal airspace sampling was performed during these operations after investigator training using a particle counter (model 8306; Particles Plus, Stoughton, Massachusetts, USA) to measure 30-s periods both at baseline (before surgical incision) and then episodically during the procedure by positioning the device’s isokinetic probe inlet 10 cm from the target area^8^. This device cumulatively measures particles by laser diode with differential counting (0.3–25 µm) at 0.1 Cubic Feet per Minute flow (2.83 Litres per Minute).

## Results

**Fig. 1:**
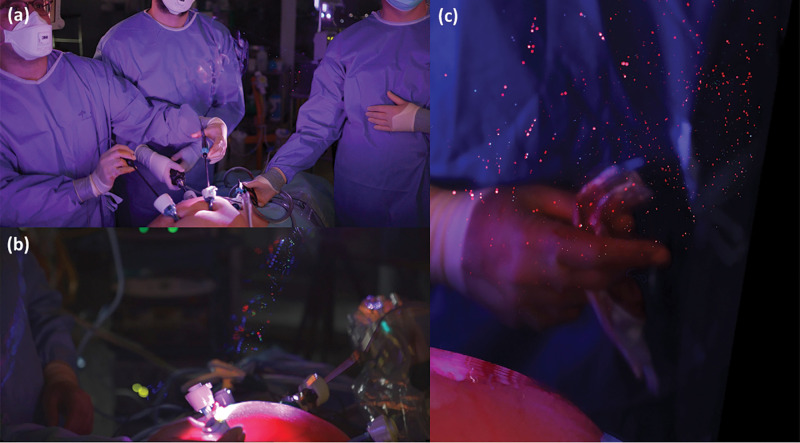
Comet plot images (maximum-averaged stack of sequential 12-bit Raw frames of intraoperative footage) showing particles including trajectories as repeated particle groups in the operative airspace during (**a**) dissection of the gallbladder from the liver bed during cholecystectomy (**b**) with the laparoscopic port luer lock open to vent intrabdominal smoke and (**c**) on specimen extraction via a periumbilical incision (imagery best appreciated by viewing the associated video).

Smoke studies revealed effective dissipation of smoke by positive-pressure room ventilation with the OR fully empty, as expected. However, during simulated operative scenarios, smoke behaviour was significantly different, with evident upwards drift from the operating site enveloping members of the surgical team (*Video 1* and *Appendix S1*). Increased crowding of the operating table with people and equipment caused increased local air stagnation. Intraoperative footage during patient operations showed smoke and particles (evidenced as scintillations) moving similarly during surgery, notable even with the instruments *in situ*, as well as during manoeuvres including trocar instrumentation, and venting and specimen removal (*Fig. 1* and *Video 1*). Aerosol and particle leakage into the OR airspace was most evident during the operative phase of intra-abdominal dissection using hook cautery.

Particle counts confirmed increasing particulate concentration after initiation of the operation, reaching extracorporeal airspace levels in excess of 1×10^6^ particles per m^3^, the majority being 0.3–0.5 µm and, during cholecystectomy, 5–10 µm (*Table 1*). Counts were particularly increased during electrocautery dissection of the gallbladder from the liver bed during cholecystectomy compared with dissection of the mesoappendix during appendicectomy, which in turn was associated with higher counts than were observed during intra-abdominal reduction of a parastomal hernia (done without cautery dissection). Trocar venting caused the highest concentration of particulate effluvium.

**Table 1:** Mean extracorporeal airspace particle counts during operations

Operative phase	Total no. of particles	Particle count by size					
		0.3 μm	0.5 μm	1.0 μm	2.5 μm	5 μm	10 μm
**Laparoscopically assisted parastomal hernia repair (*n* = 1)**							
Background before incision	126 188	60 505 (1)	33 901 (1)	9888 (1)	14 361 (1)	3295 (1)	4237 (1)
Intraoperative	120 538	68 510 (1)	36 962 (1)	3766 (0)	5650 (0)	1883 (1)	3766 (1)
Intraoperative, trocar venting (5-mm port)	289 225	130 311 (2)	73 807 (2)	30 724 (3)	31 077 (2)	13 066 (4)	10 241 (2)
**Laparoscopic appendicectomy (*n* = 2)**							
Background before incision	423 420	184 813 (1)	112 771 (1)	61 565 (1)	41 789 (1)	10 006 (1)	12 477 (1)
Intraoperative, before dissection	1 068 619	421 657 (2)	307 943 (3)	180 811 (3)	108 062 (3)	29 664 (3)	20 482 (2)
Intraoperative, during dissection	1 805 989	1 689 100 (9)	81 223 (1)	20 835 (0)	12 007 (0)	1059 (0)	1766 (0)
**Laparoscopic cholecystectomy (*n* = 3)**							
Background before incision	151 536	39 729 (1)	57 563 (1)	27 545 (1)	22 072 (1)	4307 (1)	322 (1)
Intraoperative, during dissection	776 358	300 528 (8)	11 830 (0)	2472 (0)	4414 (0)	420 139 (98)	36 977 (115)
Intraoperetive, trocar venting during dissection	9 271 468	8 289 764 (208)	127 839 (2)	5650 (0)	2118 (0)	674 726 (157)	171 371 (532)

Values in parentheses are ratio of counts in operative phase to background counts (BKD, counts after patient positioning, preparation, and draping but before incision). Dissection during appendicectomy and cholecystectomy was performed using hook monopolar point diathermy.

## Discussion

This study focused on establishing methods and indicative data regarding the operative airspace particulate contamination occurring during laparoscopy, using open procedures as control in a simulation study as well as in two common general surgical laparoscopic operations that employ electrocautery to different extents (versus a laparoscopic operation without electrocautery). This is important as many surgical teams feel any such occupational hazard to be either theoretical or mitigated anyway by room ventilation and perhaps standard surgical masks^9^. However, OR ventilation standards and indeed commissioning assume that empty theatres and surgical masks protect only primarily against large fluid droplet inhalation (and are loose fitting).

The evaluations in this study reflect actual workspace conditions of OR teams, corroborating simulation data with live intraoperative flow visualization and sensitive particle counting (necessary as the laser sheet provides only a 2D slice and so underestimates total particle concentration). Together, these show that the surgical team is exposed to considerable amounts of particles and pollutants during laparoscopy. Indeed, aerosol (containing gas and particles) leaks continuously from the patient during laparoscopic operations, with such flue comprising the constituents of the pneumoperitoneal gas including any noxious components present. The local OR airspace pollution is particularly marked during the cautery dissection phase of the operation, and occurs constantly rather than just at the time of instrument insertion and removal^10^. Particle counts increase during the operation even without cautery and trocar venting causes the greatest effluvium stream. A substantial proportion of the aerosolized particles are less than 5 µm in size and so may remain airborne indefinitely unless removed^11^. All airborne particles smaller than 10 µm can be inhaled, with those greater than 2.5 µm depositing within the nose, pharynx, trachea, and bronchi, whereas smaller particles reach the bronchioles and alveoli where those smaller than 0.1 µm are absorbed into the circulatory system^9^ .The smoke simulation studies show that modern OR positive-pressure ventilation sufficient to meet official requirements (https://www.gov.uk/government/collections/health-technical-memorandum-disinfection-and-sterilization) is not powerful enough to counteract the local airspace environment created by surgical teams carrying out their work. This means that there is relative stagnation of haze in the operative airspace above the abdomen during laparoscopic operation, with entrainment towards surgical team members likely induced by movement, body heat, and electrostaticity^12^.

Although investigation of COVID-19 infectivity owing to laparoscopic access is ongoing, the pandemic has already caused considerable reflection regarding the aerosol-generating capability of laparoscopy, and encouraged re-examination of practice and equipment from this new perspective^13^^,^^14^. Most attention has focused on surgical smoke extraction^15^^,^^16^, although to date most recommendations have been based on theoretical extrapolations^17^^,^^18^ from expert groups and industry without independent, empirical data to guide evolved thinking and practice regarding laparoscopic care^19^. Importantly, the pandemic has also educated, equipped, and enabled familiarity of OR teams with better respiratory protection (such as N95/FFP2/FFP3 masks) and smoke extraction principles and systems, including laparoscopic devices. The opportunity therefore presents to continue to protect surgical personnel in both these terms and others (including considering OR redesign). Further work is of course needed to expand this work to additional operations (including more major resectional operations of longer duration, greater energy device use, and larger-diameter instrumentation, including trocars and staplers, which likely exacerbate the levels of airspace contamination^10^) and indeed other surgical specialties.

The implications of the present study regarding aerosolization at laparoscopy extend beyond the present pandemic. The results provide insight into both mechanism and degree as well as assessment methodology for future evaluations, including those of mitigation strategies. Although practice advice^1^ and personal protective equipment^20^ have a positive impact, there should also be much confidence that improved awareness and smart engineering innovations can ameliorate current laparoscopic access equipment and environmental standards, ensuring better occupational hygiene for OR teams.

## Supplementary Material

znab114_Supplementary_Data
