# Supplementary material for: Aerosols, airflow, and airspace contamination during laparoscopy
Source: Br J Surg. 2021 Apr 8;108(9):1022–5. doi: 10.1093/bjs/znab114 (PMC8083507; doi:10.1093/bjs/znab114)

**SPECTRUM ENVIRONMENTAL LIMITED**<br>Unit 6, 117 Baldoyle Ind. Est. Dublin 13<br>Tel: 00 353 1 8398994 FAX: (01) 8398381<br>email: info@spectrumenvironmental.ie	**Customer**	Mater Public Hospital	
	**Location**		
	**System**		
	**Report No.**		

**Document Title**	**Form No.**	**Revision**	**Revision Date**	**QMS**
Report Summary and Conclusion	SE009	Rev04	22 Sep 2015	N/A

SUMMARY AND CONCLUSION

Mater Theatre 6 air pattern study

A study of smoke patterns was carried out in Theatre 6 of the Whitty wing of the Mater Public Hospital. The study was carried out initially with the ventilation off for comparison purposes and then with the ventilation system on in full running mode.

The ventilation for the theatre supplies HEPA filtered air to the theatre at a rate of 25 air changes per hour through 4 ceiling mounted supply grilles with a four way throw pattern as illustrated in the sketch below. HEPA filtration ensures a particulate free and therefore bacteria free supply air to the theatre. The high ventilation rate ensures that smoke is quickly carried away and prevents a large build-up of haze. There is a low-level extract in the corner of the theatre and the rest of the air spills out through pressure stabilisers to the anaesthetic room and the exit bay. There is a pressure cascade of 25 Pascals in the theatre through the Anaesthetic, the dirty utility and the exit bay.

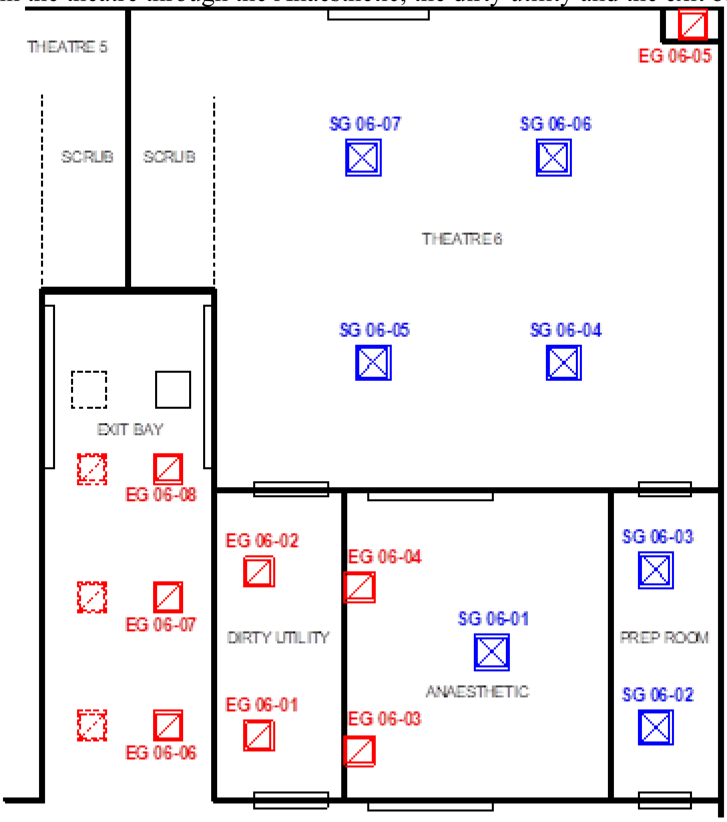

A Concept Engineering "Air Trace" smoke generator was used to generate low levels of smoke to demonstrate the airflow patterns. The generator was piped with a 25mm pipe to the point of interest. As the studies continued, the generator output was piped into a larger 100mm flexible duct to dissipate any velocity force from the generator. The larger flexible duct was abandoned, and the generator setting was set to a low output which proved adequate for smoke studies with the 25mm pipe. The piped smoke was essentially isokinetic and isothermal with little influence on the surrounding air patterns.

Ventilation off

The patterns with the ventilation off demonstrated that smoke drifted around the theatre in an uncontrolled manner and stayed in the theatre for an extended period of time. This was the case in the anaesthetic room as well.

RECORDED BY:	DATE:
REVIEWED BY:	DATE:

Ventilation on

The ventilation system circulated the smoke in the anaesthetic room and the theatre effectively and the smoke dissipated more rapidly.

Operation studies

Various operation set ups were studied with the use of a mannequin ("patient") on the table. Multiple cameras were used to record the smoke/air patterns.

The pipe from the smoke generator was placed at the surgical site and the surgical team took up their positions. Smoke was released for approximately 5 seconds for three or more studies per setup. A small extract fan was placed near to the surgical site for the latter half of the Laparotomy study and thereafter for the simulated operation setups.

The following setups were studied.

- **General setup with the team around the "patient" on the table.**
- **Intermediate e.g. Open Hernia**
- **Laparotomy**
- **Laparoscopic Appendicectomy/Cholecystectomy**
- **Laparoscopic Anterior Resection**
- **Laparoscopic Appendicectomy/Cholecystectomy**

All the studies showed that smoke drifted upwards from the surgical site and enveloped members of the surgical team. Although the theatre is well ventilated in accordance with the HTM requirements, the airflow is not powerful enough to counteract the environment created by the team in carrying out their work. A small extract fan placed near to the table had no noticeable effect on preventing the upward drift of air. It should be noted that this small extract is not specifically designed to function as an extract at close quarters.

Powered Respirators

Studies were also carried out with members of the surgical team wearing powered hood respirators. The studies showed that the exhaust from these respirators disturbed the surrounding air pattern which then circulated with the local air currents.

Photos from three video cameras illustrate the patterns along with the relevant video sequences.

Stephen Ryan

STEPHEN RYAN
Spectrum Environmental Ltd

Indicative Photographs from Smoke Studies Assessment in Operating Theatre with Positive Pressure Room Ventilation and Surgical Simulation Scenarios (25 Room Air Exchanges/hr, 25 Pa).

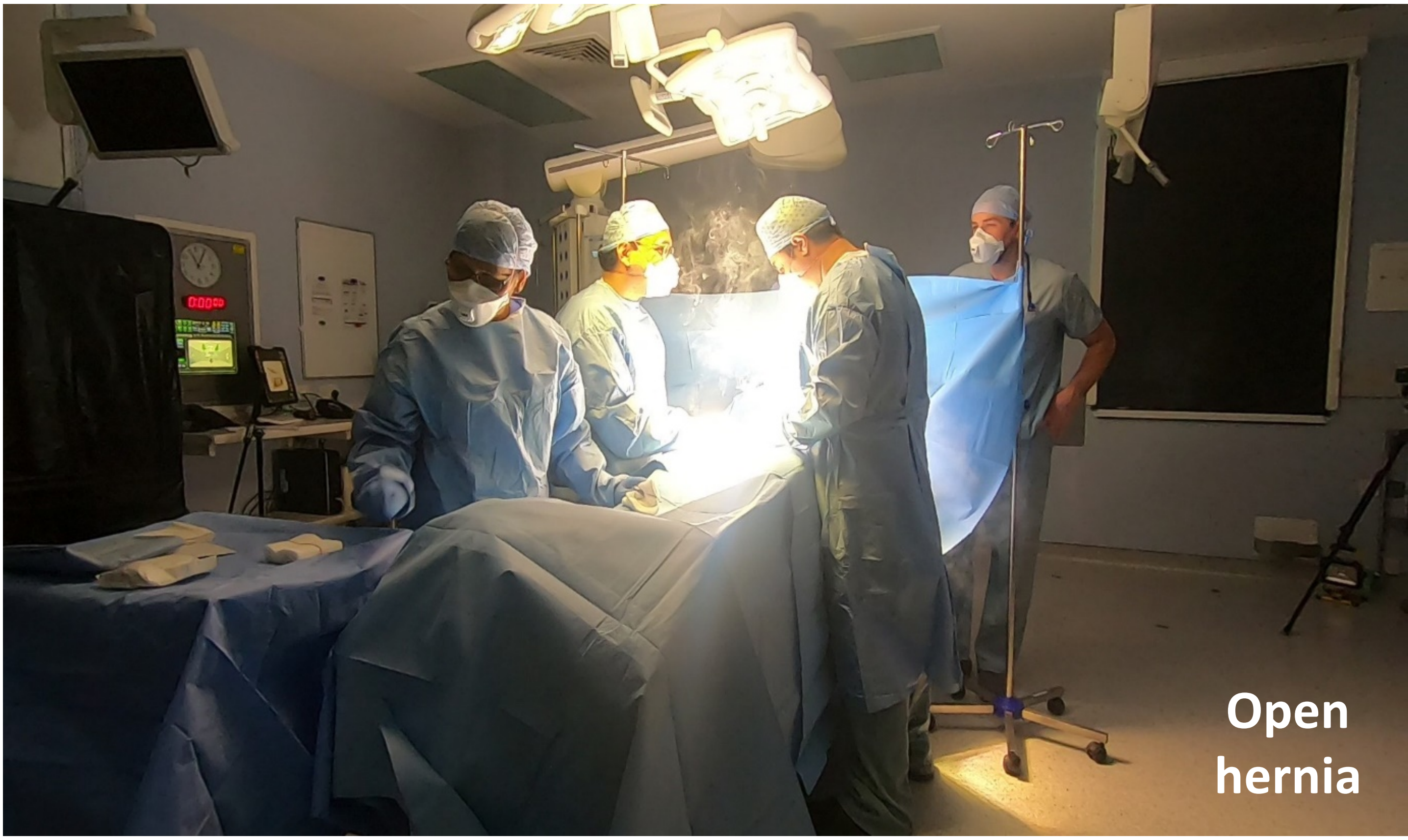

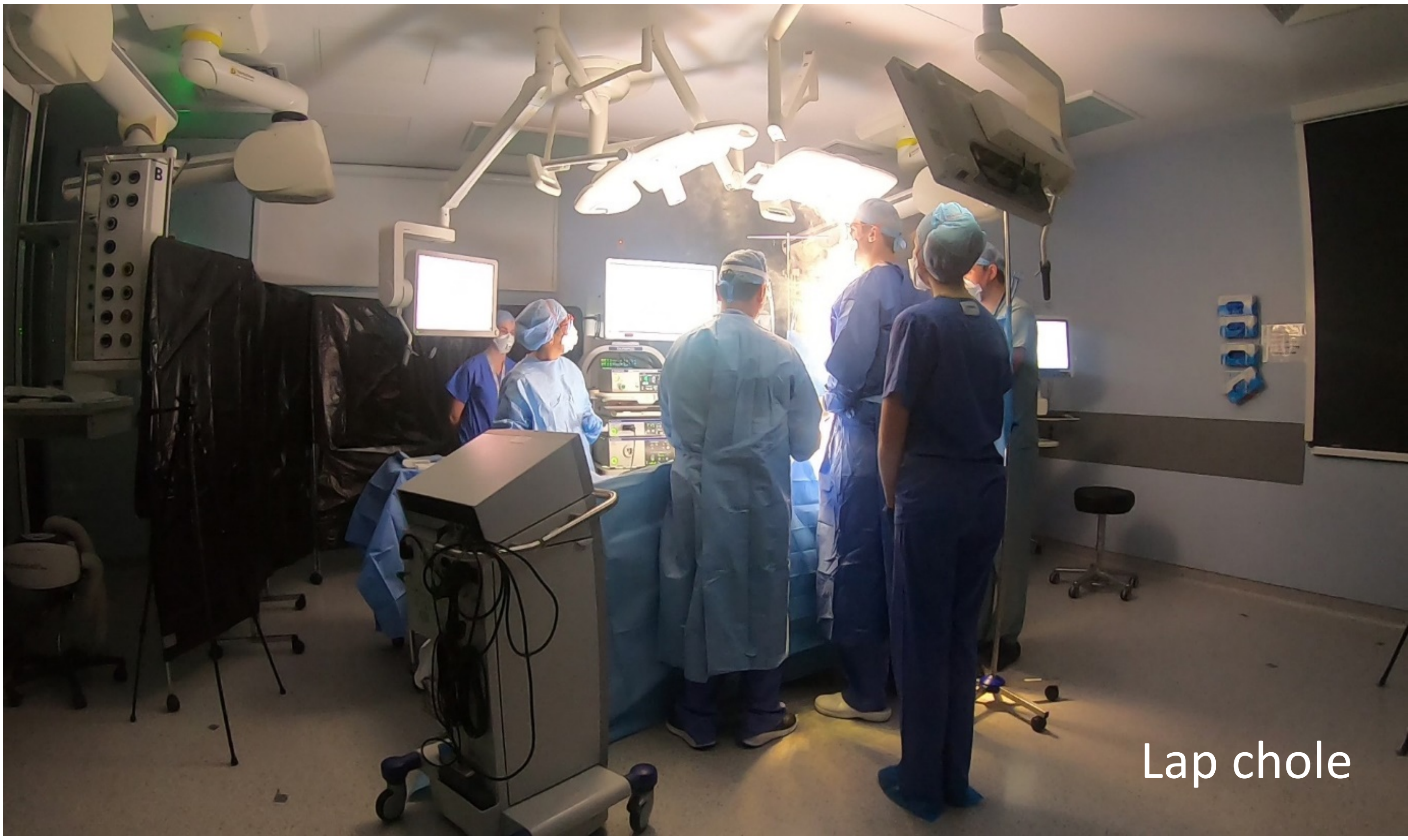

Indicative Photographs from Smoke Studies Assessment in Operating Theatre with Positive Pressure Room Ventilation and Surgical Simulation Scenarios (25 Room Air Exchanges/hr, 25 Pa).

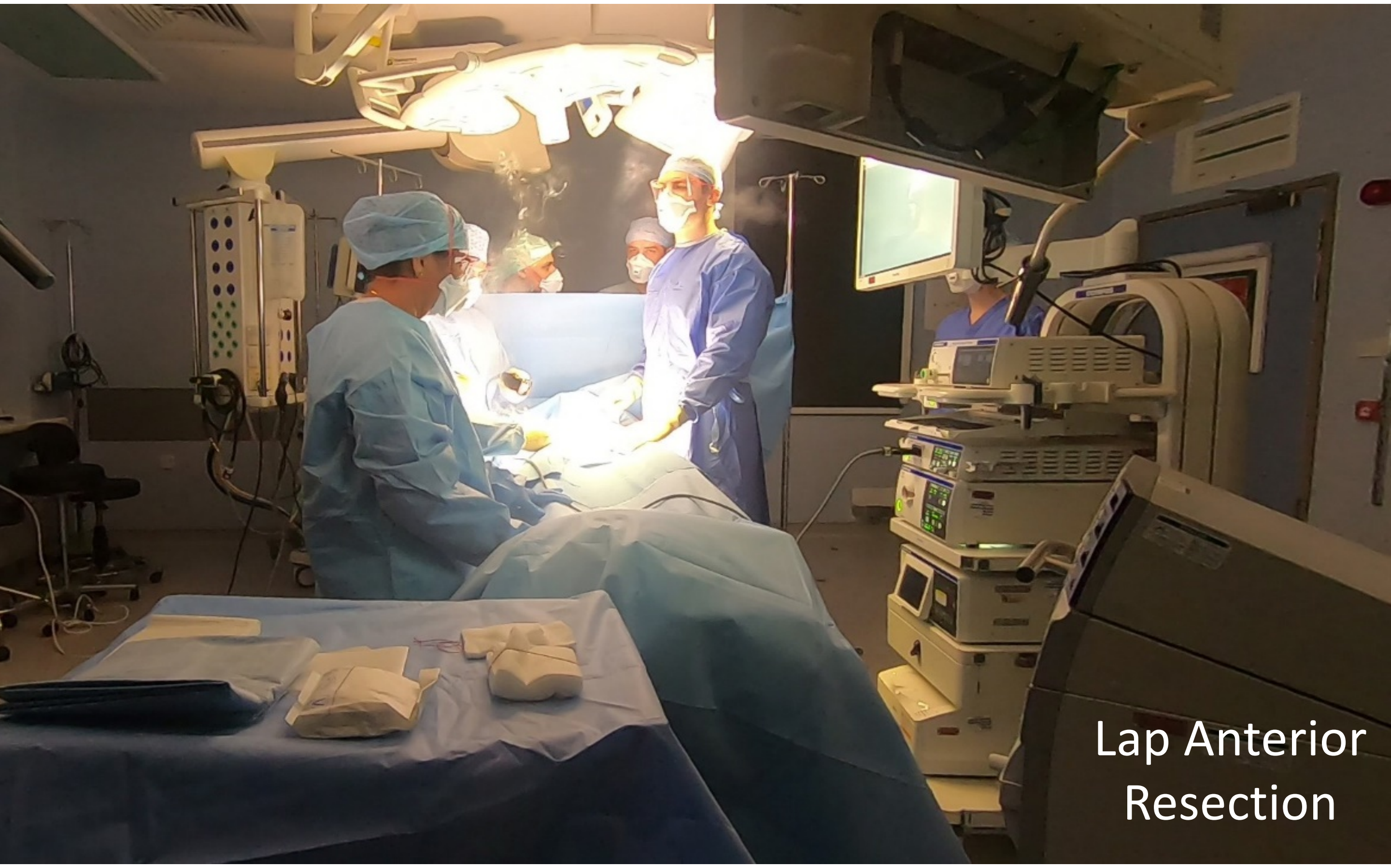

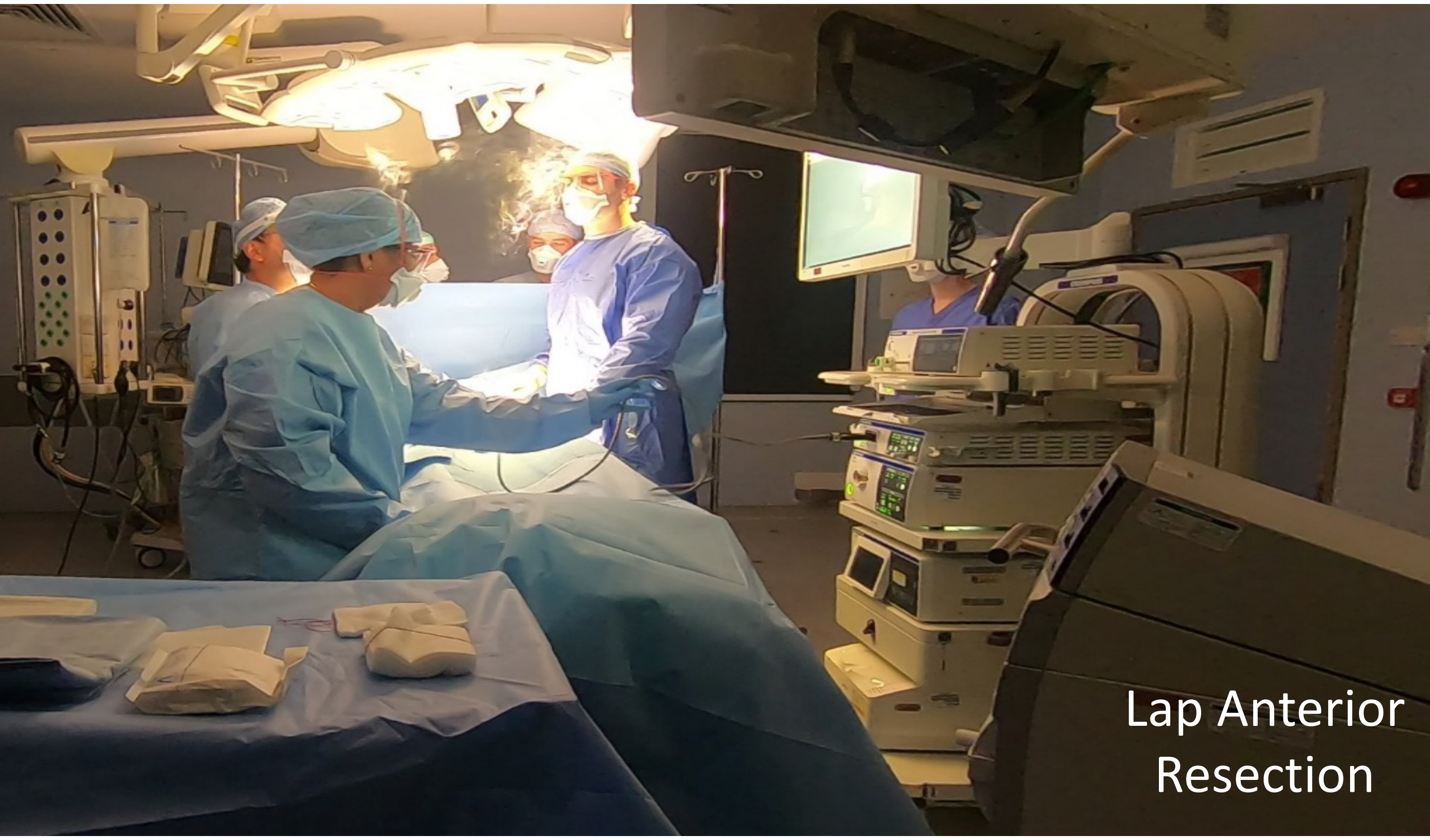

Supplement: znab114_Supplementary_Data [file znab114_Supplementary_Data.pdf]
